# The association of dietary resistance starch intake with all-cause and cause-specific mortality

**DOI:** 10.3389/fnut.2022.1004667

**Published:** 2022-12-08

**Authors:** Jiang Wan, Xiaocong Li, Ming Gu, Qi Li, Chuyun Wang, Run Yuan, Lin Li, Xiang Li, Shaodong Ye, Jichun Chen

**Affiliations:** ^1^Department of Epidemiology, Key Laboratory of Cardiovascular Epidemiology, Fuwai Hospital, National Center for Cardiovascular Diseases, Chinese Academy of Medical Sciences and Peking Union Medical College, Beijing, China; ^2^Medical Research and Biometrics Center, National Clinical Research Center for Cardiovascular Diseases, Fuwai Hospital, National Center for Cardiovascular Diseases, Peking Union Medical College and Chinese Academy of Medical Sciences, Beijing, China; ^3^Department of Cardiac Surgery, Fuwai Hospital, National Center for Cardiovascular Diseases, Chinese Academy of Medical Sciences and Peking Union Medical College, Beijing, China; ^4^Department of Nutrition, Fuwai Hospital, National Center for Cardiovascular Diseases, Chinese Academy of Medical Sciences and Peking Union Medical College, Beijing, China; ^5^Department of Cardiology, Fuwai Hospital, National Center for Cardiovascular Diseases, Chinese Academy of Medical Sciences and Peking Union Medical College, Beijing, China

**Keywords:** dietary, resistant starch, mortality, CVD, cardiovascular disease, cancer

## Abstract

**Background:**

Several studies have estimated daily intake of resistant starch (RS), but no studies have investigated the relationship of RS intake with mortality.

**Objective:**

We aimed to examine associations between RS intake and all-cause and cause-specific mortality.

**Methods:**

Data from US National Health and Nutrition Examination Survey (NHANES) from 1999 to 2018 with 24-h dietary recall data was used in current study. The main exposure in this study was RS intake, and the main outcome was the mortality status of participants until December 31, 2019. The multivariable Cox proportional hazards regression models were developed to evaluate the hazard ratios (HRs) and 95% confidence interval (95% CI) of cardiovascular disease (CVD), cancer, and all-cause mortality associated with RS intake.

**Results:**

A total of 42,586 US adults [mean (SD) age, 46.91 (16.88) years; 22,328 (52.43%) female] were included in the present analysis. During the 454,252 person-years of follow-up, 7,043 all-cause deaths occurred, including 1,809 deaths from CVD and 1,574 deaths from cancer. The multivariable-adjusted HRs for CVD, cancer, and all-cause mortality per quintile increase in RS intake were 1 (95%CI, 0.97–1.04), 0.96 (95%CI, 0.93–1), and 0.96 (95%CI, 0.95–0.98), respectively. The associations remained similar in the subgroup and sensitivity analyses.

**Conclusion:**

Higher RS intake is significantly associated with lower cancer and all-cause mortality, but not significantly with CVD mortality. Future studies focusing on other populations with different food sources of RS and RS subtypes are needed to access the dose–response relationship and to improve global dietary recommendations.

## 1. Introduction

Diet plays a crucial role in people's overall health and well-being. Previous studies have identified dietary factors associated with mortality (1, 2). Suboptimal diet, an important preventable risk factor for non-communicable diseases (NCDs), is responsible for more deaths than any other risks worldwide (3), and improvement of diet could potentially prevent one in every five deaths globally (4).

Carbohydrates are the main source of energy for most of the world's population, providing 50% or more of daily energy (5). There are already evidences that high-carbohydrate diets increase the risk of mortality (6). Beyond the quantity, the quality, and food sources of carbohydrate have been proved to play a role in health consequences (7). Starch is the major source of carbohydrate in the human diet, and resistance starch (RS) is defined as the total amount of starch and its degradation products that resists digestion in the small intestine of healthy individuals. Meeting the three criteria for being a prebiotic (8): resistance to the upper gastrointestinal environment, fermentation of the gut microbiota, and selective stimulation of beneficial bacterial growth and/or activity, resistant starch (RS) seems to be a promising nutritional strategy to improve people's health. There is limited evidence that RS can benefit gut health (9), glucose homeostasis (10), insulin sensitivity (11), lipid profile (12), cancer (13), chronic kidney disease (14), and improve inflammation and oxidative stress (15).

To our knowledge, no studies have assessed the associations of RS intake with mortality. Given the potential benefits of RS and the uncertainty in the literature, we conducted the current research to examine associations between RS intake and all-cause and cause-specific mortality using the data of U.S. adults from the US National Health and Nutrition Examination Survey (NHANES).

## 2. Materials and methods

### 2.1. Study design and population

The National Health and Nutrition Examination Survey (NHANES) is a stratified, multistage study designed by the National Center for Health Statistics (NCHS) to assess the health and nutritional status among a nationally representative sample. All data and materials used in this study from the NHANES database are free and directly downloadable from https://wwwn.cdc.gov/nchs/nhanes/Default.aspx. Mortality data are available from https://ftp.cdc.gov/pub/Health_Statistics/NCHS/datalinkage/linked_mortality/.

**Figure 1:**
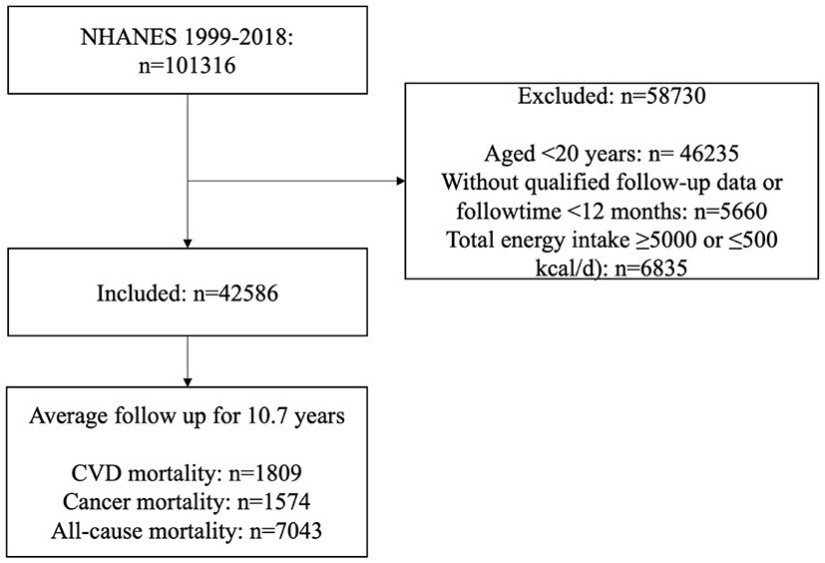
Flow diagram of inclusion and exclusion. CVD, cardiovascular disease; NHANES, National Health and Nutrition Examination Survey.

To maximize the sample size, datasets required for this study during the 10 cycles from 1999 to 2018 were downloaded. Finally, 42,586 participants (including 20,258 males and 22,328 females, aged ≥20 years) were enrolled with qualified follow-up data (without any missing information on mortality) and dietary data (without any missing information on any dietary intake, and total energy intake ≥5,000 or ≤500 kcal/d). A flow diagram for the inclusion and exclusion of participants in this study is presented in Figure 1. The institutional review board approval of the National Center for Health Statistics (NCHS) and written informed consent for each participant were obtained before data collection.

### 2.2. Main exposure and resistant starch assessment

The main exposure in this study was RS intake. The 24-h dietary recall was employed to collect the food intake data for two non-consecutive days. The first 24-h dietary recall was conducted manually, and the second dietary recall (added in 2002 and later) was collected by telephone and was scheduled 3- to 10-days later (16). To assess RS consumed by NHANES participants, the weighted average of RS for each food in the RS database was first matched with the unique eight-digit Food and Nutritious Database for Dietary Studies (FNDDS) food code (Supplementary 1) (17, 18), which defined food groups and subgroup. Next, the amount of RS in each food record was calculated by multiplying the weighted average by the grams of the food. Finally, the amount of RS in each food record was added up to get the total amount of RS consumed by each individual. Data are reported as total RS in g/(d * 1,000 kcal).

### 2.3. Main outcome

The main outcome of this study was the survival condition of participants, which has been updated with mortality follow-up data by the National Death Index (NDI) through December 31, 2019. As the most complete source of death information in the United States, the NDI has been used to determine the mortality status of participants in this study. The International Classification of Diseases, Tenth Edition (ICD-10) was used to determine the specific cause of death. The ICD-10 codes for CVD were I00–I09, I11, I13, and I20–I51. The ICD-10 codes for cancer were C00–C97. In the end, 7,043 people died, including 1,809 from cardiovascular disease (CVD) and 1,574 from cancer.

### 2.4. Covariates

Non-dietary covariates included age (years), sex (male/female), race/ethnicity (Mexican American/Other Hispanic/Non-Hispanic White/Non-Hispanic Black/Other Race—including Multi-Racial), educational level (less than high school/high school diploma—including General Educational Development/college or above), body mass index (kg/m^2^), smoking status (never smoked/currently smoking/ex-smoking), drink status (yes/no), disease histories of hypertension, dyslipidemia, and diabetes mellitus (yes/no), marriage status (yes/no), and poverty income ratio (PIR) (<1.3/1.3–3.49/≥ 3.5). Body mass index (BMI) was calculated as weight in kilograms divided by height in meters squared. Participants who smoked at least 100 cigarettes during their lifetime were classified as smokers, and drinkers were defined as individuals who drank a minimum of 12 drinks in any given year. Participants could be defined as comorbid conditions (cancer, CVD, diabetes, hypertension, and dyslipidemia) if they reported that they had been told by a health care professional that they had these conditions and/or were taking prescription drugs for them and/or met the appropriate diagnostic criteria.

### 2.5. Statistics analysis

According to the NHANES analytic guidelines, sample weights, stratification, clustering were taken into consideration to account for the complex, multistage, probability sampling survey design. Data years were combined (using different sampling weights) to maximize sample sizes and evaluate for time trends. Demographic characteristics, dietary intakes, examination variables, and laboratory variables were presented as mean ± SD for continuous variables and as number (percentage) for categorical variables. χ2 tests and one-way analyses of variance were applied to compare the differences of baseline characteristics and mortality status by quantiles. The Bonferroni test was used for multiple comparisons.

The multivariable Cox proportional hazards regression models were developed to evaluate the hazard ratios (HRs) and 95% confidence interval (95% CI) of CVD, cancer, and all-cause mortality associated with RS intake. We first assessed the proportional hazards assumption by evaluating the weighted Schoenfeld residuals (19), and several violations were observed (*P* < 0.05). The violation of proportional risk assumption is addressed by adding its interaction with time to the model. Survival time was calculated as the number of months from the date of NHANES interview until death or the date of census (December 31, 2019). To control the potential confounders, age, sex, race/ethnicity were adjusted in model 1. We further adjusted for carbohydrate intake, educational level, smoking, drinking, history of relevant disease in model 2. In our final model 3, disease histories of diabetes mellitus, hypertension, and dyslipidemia, marriage status, and income were adjusted. Resistant starch intake was first fitted as an unweighted restricted cubic spline (RCS) with four knots at 5^*th*^, 35^*th*^, 65^*th*^, and 95^*th*^ centiles and then divided into quintiles to flexibly model the association of RS intake with mortality. A 20-percentile increase was used to estimate the HRs for mortality from CVD, cancer, and all-cause. The trends were estimated by treating the quintiles as a continuous variable. Interaction between continuous linear quintiles of RS intake and covariates was tested by introducing a two-factor interaction term in the multivariable adjusted Cox regression model. Participants with missing values are not included in the corresponding model.

Based on previous evidence of possible effect modification, we conducted subgroup analyses for associations between RS intake and mortality according to several confounding factors at baseline. We conducted several sensitivity analyses to test the robustness of our findings. First, we excluded participants who were followed for less than 5 years or died within 5 years. Second, we excluded the participants with a history of CVD or cancer. Third, we conducted a competing risk model to evaluate and quantify the bias of competing risks. Fourth, due to the lack of some variables for calculating HEI-2015 (Healthy Eating Index) in NHANES from 1999 to 2004, another sensitivity analysis was carried out by incorporating HEI-2015 into the model using the data of NHANES from 2005 to 2018. Fifth, a newly defined CVD outcome with ICD-10 codes I00–I09, I11, I13, I20–I51, and I60–I69 was included in the analysis.

All statistical analyses were performed with SAS 9.4 (SAS Institute Inc., Cary, NC, USA) and R 4.1.1 (R Core Team, Vienna, Austria). The two-sided *P*-values <0.05 were considered statistically significant.

## 3. Results

### 3.1. Baseline characteristics

For the 42,586 US adults included in the present analysis, mean (SD) age at baseline was 46.91 (16.88) years and 22,328 (52.43%) of all participants were female. The mean ± SD and median (interquartile range) follow-up was 11.00 ± 5.18 and 10.25 (8.33) years, respectively. During the 454,252 person-years of follow-up, 7,043 all-cause deaths occurred, including 1,809 deaths from CVDs and 1,574 deaths from cancer. Table 1 shows the characteristics of the participants at baseline according to the quintiles of RS intake. Compared with participants with the lowest RS intake, participants with the highest RS intake were more likely to be older, female, married, non-drinkers, non-smokers, Mexican American and Hispanic; to have higher SBP, educational level; and to have lower DBP and BMI. And the proportion of participants with morbidity conditions (diabetes, hypertension, dyslipidemia, and CVD) increased and the proportion of participants with cancer decreased.

**Table 1:** Characteristics of study participants according to quintiles of resistant starch intake.

**Characteristic^a^**	**Total**	**Quintiles of resistant starch intake**					***P*-value^b^**
		**Quintile 1**	**Quintile 2**	**Quintile 3**	**Quintile 4**	**Quintile 5**	
Participants, No.	42,586	8,518	8,516	8,515	8,516	8,521	NA
Age, years	46.91 ± 16.88	45.77 ± 16.24	47.13 ± 16.90	46.52 ± 16.87	47.22 ± 17.12	48.15 ± 17.25	<0.001
Female	22,328 (52.43)	4,425 (51.94)	4,472 (52.51)	4,314 (50.66)	4,479 (52.6)	4,678 (54.9)	<0.001
Followtime, years	11.00 ± 5.18	12.00 ± 5.10	11.29 ± 5.14	10.83 ± 5.13	10.55 ± 5.16	10.11 ± 5.18	<0.001
SBP, mm Hg^c^	122.32 ± 17.74	121.87 ± 17.42	122.42 ± 17.93	122.23 ± 17.09	122.34 ± 17.92	122.86 ± 18.42	<0.001
DBP, mm Hg^c^	70.73 ± 12.39	71.11 ± 12.45	70.91 ± 12.50	70.91 ± 12.53	70.41 ± 12.13	70.18 ± 12.30	<0.001
BMI, kg/m^2^^c^	28.67 ± 6.69	28.56 ± 6.69	28.73 ± 6.62	28.80 ± 6.83	28.77 ± 6.76	28.46 ± 6.48	0.001
PIR^c^							<0.001
<1.3	11,141 (26.16)	2,380 (27.94)	2,175 (25.53)	2,031 (23.86)	2,124 (24.94)	2,467 (28.96)	
1.3–3.49	14,381 (33.77)	2,925 (34.33)	2,864 (33.63)	2,867 (33.68)	2,904 (34.1)	2,810 (32.97)	
≥3.5	17,064 (40.07)	3,213 (37.72)	3,477 (40.83)	3,616 (42.47)	3,488 (40.96)	3,244 (38.07)	
Smoking							<0.001
Never smoked	22,337 (52.45)	3,889 (45.65)	4,307 (50.57)	4,520 (53.08)	4,749 (55.76)	4,995 (58.62)	
Currently smoking	9,234 (21.68)	2,578 (30.27)	1,990 (23.37)	1,747 (20.51)	1,500 (17.61)	1,286 (15.1)	
Ex-smoking	11,015 (25.86)	2,051 (24.08)	2,219 (26.06)	2,249 (26.41)	2,268 (26.63)	2,240 (26.28)	
Drinking	30,375 (71.33)	6,275 (73.67)	6,214 (72.97)	6,186 (72.64)	6,012 (70.6)	5,591 (65.62)	<0.001
Educational level							<0.001
Less than high school	7,522 (17.66)	1,579 (18.54)	1,335 (15.68)	1,384 (16.25)	1,458 (17.12)	1,824 (21.41)	
High school diploma or GED^c^	10,067 (23.64)	2,259 (26.52)	2,118 (24.87)	1,914 (22.48)	1,982 (23.28)	1,736 (20.37)	
College or above	24,997 (58.7)	4,680 (54.94)	5,063 (59.45)	5,217 (61.27)	5,076 (59.61)	4,961 (58.22)	
Married	23,923 (56.17)	4,416 (51.85)	4,788 (56.22)	4,886 (57.38)	4,929 (57.88)	4,944 (58.03)	<0.001
Race/ethnicity							<0.001
Mexican American	3,407 (8)	413 (4.85)	500 (5.87)	625 (7.35)	885 (10.39)	1,073 (12.59)	
Other Hispanic	2,299 (5.4)	383 (4.5)	367 (4.31)	429 (5.03)	436 (5.12)	735 (8.63)	
Non-Hispanic White	29,619 (69.55)	6,046 (70.98)	6,353 (74.6)	6,137 (72.08)	5,827 (68.43)	5,088 (59.72)	
Non-Hispanic Black	4,633 (10.88)	1,181 (13.87)	925 (10.86)	863 (10.14)	807 (9.48)	829 (9.73)	
Other Race	2,628 (6.17)	495 (5.81)	371 (4.35)	460 (5.41)	561 (6.59)	796 (9.34)	
Diabetes	5,160 (12.12)	839 (9.85)	961 (11.28)	982 (11.53)	1,047 (12.29)	1,331 (15.62)	<0.001
Hypertension	17,939 (42.12)	3,575 (41.97)	3,625 (42.56)	3,488 (40.96)	3,555 (41.74)	3,697 (43.39)	0.024
Dyslipidemia	13,166 (30.92)	2,412 (28.32)	2,684 (31.52)	2,575 (30.24)	2,677 (31.43)	2,818 (33.07)	<0.001
History of CVD^c^	4,583 (10.76)	927 (10.88)	989 (11.61)	828 (9.72)	899 (10.56)	940 (11.03)	0.002
History of cancer	3,888 (9.13)	758 (8.9)	867 (10.18)	759 (8.91)	799 (9.38)	705 (8.27)	<0.001

a Values are means ± SDs for continuous variables and numbers (%) for categorical variables.

b *P*-value for the comparisons between quintiles.

c RS, resistant starch; SBP, systolic blood pressure; DBP, diastolic blood pressure; BMI, body mass index; PIR, poverty income ratio; GED, General Educational Development; CVD, cardiovascular disease.

### 3.2. RS intake and mortality

**Figure 2:**
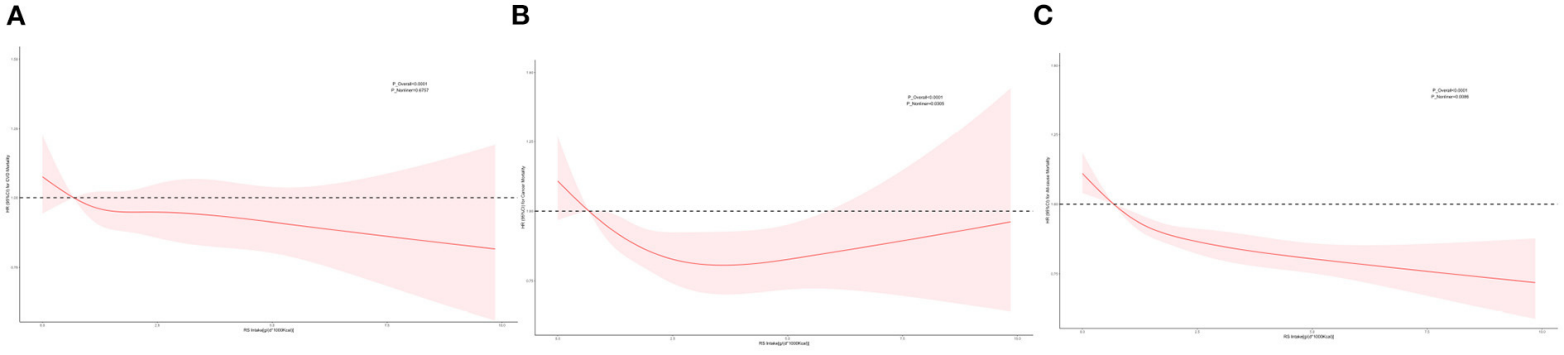
Restricted cubic spline (RCS). Multivariable-adjusted HRs (red lines) and 95%CI (pink areas) for risk of mortality in model 3. An intake of 0.68 g/(d * 1,000 kcal) was set as reference (dashed lines) (HR = 1.00). **(A)** RCS for CVD mortality. **(B)** RCS for cancer mortality. **(C)** RCS for all-cause mortality.

We used unweighted RCS to flexibly model and visualize the relationship of predicted RS intake with mortality (Figure 2). Dose–response relationship between cancer mortality risk and RS intake approximates a U-shaped curve. The risk of all-cause mortality decreased with increased RS intake, and a significant protective effect was observed at higher RS intake. Restricted cubic splines revealed possible linear or non-liner relationships of RS intake with cancer and all-cause mortality (*P*-overall <0.001 and *P*-overall <0.001, respectively; *P*-non-liner = 0.03 and *P*-non-liner <0.01, respectively).

The RS intake was associated with cancer and all-cause deaths, but not associated with CVD deaths. The multivariable-adjusted HRs for CVD mortality from the lowest quintile to the highest quintile were 1 (reference), 0.88 (95%CI, 0.76–1.02), 0.9 (95%CI, 0.77–1.04), 0.99 (95%CI, 0.85–1.15), 0.96 (95%CI, 0.83–1.12) (*P* = 0.80 for trend); for cancer mortality, 1 (reference), 0.91 (95%CI, 0.78–1.06), 0.91 (95%CI, 0.78–1.06), 0.84 (95%CI, 0.71–0.98), 0.86 (95%CI, 0.74–1.02) (*P* = 0.04 for trend); and for all-cause mortality, 1 (reference), 0.89 (95%CI, 0.83–0.96), 0.87 (95%CI, 0.81–0.94), 0.85 (95%CI, 0.79–0.92), 0.85 (95%CI, 0.78–0.91) (*P* <0.001 for trend) (Table 2).

**Table 2:** Associations between resistant starch intake and mortality.

**Characteristic**	**Quintiles of resistant starch intake**					***P*-value**	**Per quintile increase**
	**Quintile 1**	**Quintile 2**	**Quintile 3**	**Quintile 4**	**Quintile 5**	
Participants, No.	8,518	8,516	8,515	8,516	8,521	NA	NA
Followtime, years	11.46 ± 5.07	10.99 ± 5.08	10.60 ± 5.08	10.34 ± 5.11	9.94 ± 5.08	NA	NA
RS intake, g/(d * 1,000 kcal)^d^	0.33 ± 0.22	0.99 ± 0.19	1.69 ± 0.22	2.57 ± 0.32	4.74 ± 1.64	NA	NA
CVD deaths, No.^d^	391	368	337	358	355	NA	NA
Cancer deaths, No.	367	346	298	280	283	NA	NA
All-cause deaths, No.	1,580	1,510	1,324	1,305	1,324	NA	NA
**HRs (95% CI) of CVD mortality^d^**							
Model 1^a^	1 (Reference)	0.82 (0.71–0.94)	0.81 (0.7–0.94)	0.87 (0.75–1)	0.87 (0.75–1)	0.171	0.98 (0.94–1.01)
Model 2^b^	1 (Reference)	0.89 (0.77–1.02)	0.89 (0.77–1.03)	0.97 (0.84–1.13)	0.96 (0.83–1.11)	0.938	1 (0.97–1.04)
Model 3^c^	1 (Reference)	0.88 (0.76–1.02)	0.9 (0.77–1.04)	0.99 (0.85–1.15)	0.96 (0.83–1.12)	0.799	1 (0.97–1.04)
**HRs (95% CI) of cancer mortality^d^**							
Model 1^a^	1 (Reference)	0.86 (0.74–1)	0.8 (0.69–0.94)	0.76 (0.65–0.89)	0.77 (0.65–0.9)	<0.001	0.93 (0.9–0.97)
Model 2^b^	1 (Reference)	0.9 (0.78–1.05)	0.88 (0.75–1.02)	0.83 (0.71–0.97)	0.85 (0.73–1)	0.024	0.96 (0.93–0.99)
Model 3^c^	1 (Reference)	0.91 (0.78–1.06)	0.91 (0.78–1.06)	0.84 (0.71–0.98)	0.86 (0.74–1.02)	0.04	0.96 (0.93–1)
**HRs (95% CI) of All-cause mortality^d^**							
Model 1^a^	1 (Reference)	0.85 (0.79–0.91)	0.8 (0.74–0.86)	0.79 (0.73–0.85)	0.8 (0.74–0.86)	<0.001	0.95 (0.93–0.96)
Model 2^b^	1 (Reference)	0.89 (0.83–0.96)	0.86 (0.8–0.93)	0.86 (0.8–0.93)	0.86 (0.8–0.93)	<0.001	0.97 (0.95–0.98)
Model 3^c^	1 (Reference)	0.89 (0.83–0.96)	0.87 (0.81–0.94)	0.85 (0.79–0.92)	0.85 (0.78–0.91)	<0.001	0.96 (0.95–0.98)

a Cox proportional hazard model adjusted for age, sex, race/ethnicity.

b Further adjusted for total carbohydrate intake, educational level, smoking, drinking, history of CVD or cancer.

c Further adjusted for disease histories of diabetes mellitus, hypertension and dyslipidemia, marriage status, and income.

d RS, resistant starch; CVD, cardiovascular disease; HR, hazard ratio; CI, confidence interval; NA, not applicable.

A per 20-percentile increase in RS intake was not significantly associated with the risk of CVD mortality (HR, 1; 95%CI, 0.97–1.04), whereas a per 20-percentile increase in RS intake was associated with an 4% lower risk of cancer mortality (HR, 0.96; 95%CI, 0.93–1) and a 4% lower risk of all-cause mortality (HR, 0.96; 95%CI, 0.95–0.98).

### 3.3. Subgroup and sensitivity analyses

**Figure 3:**
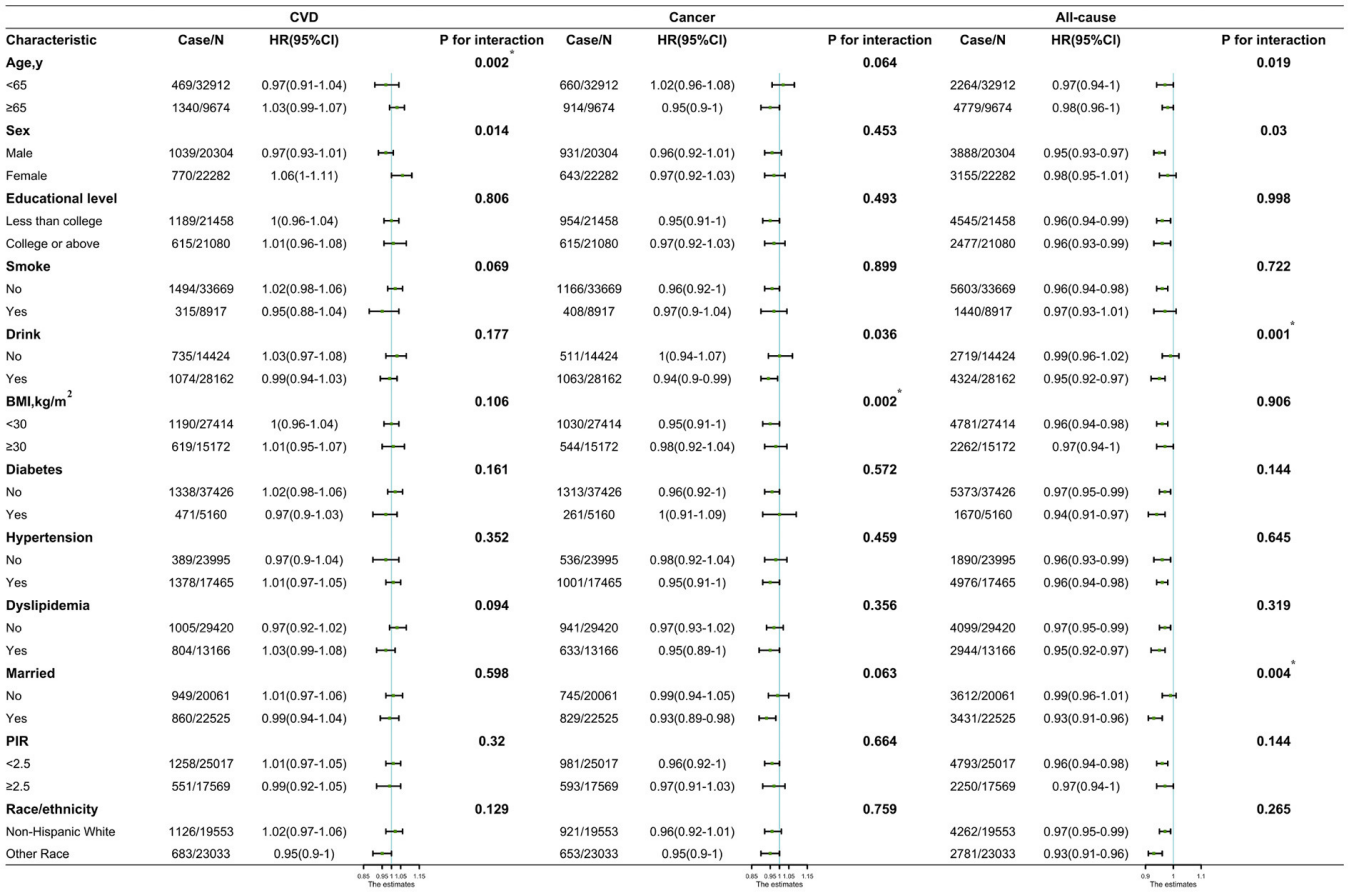
Hazard ratios (HRs) of CVD, cancer, and all-cause mortality per quintile increase in resistant starch intake by subgroups and sensitivity analyses. CVD, cardiovascular disease; HR, hazard ratio; CI, confidence interval; BMI, body mass index. *Being significant after Bonferroni correction.

In subgroup analyses, the associations between RS intake and cancer and all-cause deaths remained persistent in most subgroups (Figure 3). A statistically significant interaction between RS intake and BMI (*P* = 0.002 for interaction) for cancer. Significant interactions were found between RS intake and alcohol consumption and marital status (*P* = 0.001 and *P* = 0.004 for interaction, respectively). The HRs for cancer mortality per 20-percentile RS increase were 0.95 (95%CI, 0.91–1) among participants with BMI <30 kg/m^2^ vs. 0.98 (95%CI, 0.92–1.04) among participants with BMI ≥ 30 kg/m^2^; and for all-cause mortality, 0.95 (95%CI, 0.92–0.97) among participants who drank alcohol vs. 0.99 (95%CI, 0.96–1.02) among participants who did not drink alcohol and 0.93 (95%CI, 0.91–0.96) among participants who were married vs. 0.99 (95%CI, 0.96–1.01) among unmarried participants.

When we further excluded participants with a follow-up of less than 5 years, these associations remained similar in sensitivity analyses. The results for the newly defined CVD outcome were consistent with those for CVD in the primary analysis. Statistically significant associations were not detected when we applied a Fine-Gray competing risk model or a model with HEI-2015 or excluded patients with CVD or cancer. Details of the sensitivity analysis are shown in Supplementary 2.

## 4. Discussion

### 4.1. Main findings

To the best of our knowledge, our research is the first cohort study to investigate the association of RS intake with overall and cause-specific mortality. The estimated intake of RS in our research [2.09 ± 1.76 g/(d * 1,000 kcal)] was similar to previous studies (17). The usual average intake of RS in American adults is approximately 4.2 g/d, much less than the 15–20 g/d of RS recommended for health benefits (20). We observed that higher RS intake was associated with lower cancer and all-cause mortality in a nationally representative sample of US adults. The risk of death from cancer and any causes were 14% and 15% lower in those reporting the highest RS intake, respectively. Results from RCSs showed that individuals in the general population with an intake of RS of approximately 3 g/(d * 1,000 kcal) had the lowest risk of cancer.

A growing body of literature demonstrates that the use of dietary fiber can manipulate the microbiota and greatly impact health. Resistant starch shares some characteristics with dietary fiber and may have similar health effects, and prebiotic-RS seems to be a promising nutritional strategy (9, 15, 21). Our results were consistent with previous observational studies reporting a positive association between dietary fiber intake and health outcomes (22–25). In contrast to these findings, no evidence that RS supplementation at 30 g/day has an effect on development of colorectal cancer in carriers of hereditary colorectal cancer was found in CAPP2 study with 937 participants who were followed for up to 4 years, a randomized trial to assess the effectiveness of RS supplement on carcinoma in human beings (13). The results of these studies may be controversial depending on the tumor type, region, or ethnicity studied.

In addition, although higher dietary fiber intake was reported to be associated with a significantly reduced risk of first stroke (26), we failed to find a significant association between RS intake and CVD mortality. The role of dietary fiber in the prevention of CVD remains controversial. We speculate that it may be the result of low RS intake and low adherence, since short-term high-RS diets do not improve markers of cardiometabolic health (12). Another possibility is that increased RS intake is accompanied by increased carbohydrate intake, which increases the risk of CVD (7). Perhaps the ratio of RS to starch affected the primary outcome, which requires further in-depth study.

### 4.2. Interpretations of our findings

Several possible mechanisms could be involved in the associations of RS intake with mortality. Obesity is associated with comorbidities such as diabetes, CVD, and cancer, which are among the leading causes of death in the Western world, and RS has many properties that could ameliorate the impact of these comorbidities by promoting weight loss and/or weight maintenance (20). Consumption of RS can not only increase intestinal satiety peptide release, reduce postprandial glucose and insulin (10), but also increase fat oxidation, reduce fat storage in adipocytes, and maintain lean body mass. In addition, total energy consumption increases due to the fiber-like properties of RS, which increases the thermal effect of the food (27). Outside of these properties, RS has other notable health benefits. It has been confirmed that RS positively regulates the gut microbiome, and significantly increases stool output and fecal moisture content, as well as the concentration of short-chain fatty acids (SCFA) (28, 29). The major SCFA are acetate, propionate, and butyrate, which are primarily derived from fermentation of dietary fibers and play key roles in host gut, metabolic, and immune function mainly due to their impact on gene regulation (30). Gut microbiota actively communicates with host cells through the production of SCFA and strongly modulate multiple cellular mechanisms (30), such as regulating cell proliferation and differentiation by inducing apoptosis in colorectal cancer cells while providing energy for normal colonocytes, a situation termed the “Butyrate Paradox” (31). In addition, RS has positive effects on other functions (inflammation, cholesterol, gut hormonal activity, etc.) through bacterial fermentation in the intestine (12, 15).

### 4.3. Strengths and limitations

The strengths of this study should be acknowledged. For this study, a nationally representative sample of U.S. adults with a longitudinal study design was used to collect dietary and health data using validated methods. We first conducted this study to explore the relationship of RS intake with mortality.

However, there are several limitations. First, the amount of RS in food varies depending on how it was handled and how long it was stored. Due to the limited capabilities to quantify the actual amount of RS in these foods, RS intake may be mis-estimated resulting in erroneous results. Second, RS has been categorized into four main types, but not all RSs behave the same. In the present study, we failed to further investigate the effect of RS subtypes on health. Perhaps one of the subtypes of RS has a major effect on health outcomes, or perhaps different subtypes of RS have a combined effect on health outcomes. Third, due to methodological limitations, we failed to consider weights in RCS. Although such results reflect the NHANES population, they may not reflect the actual situation in the U.S. population.

### 4.4. Clinical importance

Some guidelines recommend that adults consume 15–20 g of RS per day for health benefits (20), however, RS intake increases with total carbohydrate intake, which increases the risk of mortality (32). Our findings suggest that total energy intake or total carbohydrate intake should be considered when increasing RS intake. Resistant starch is found naturally in several foods, the best sources being whole grains and legumes. We suggest taking more foods rich in RS to increase daily RS intake without increasing total energy intake or total carbohydrate intake. In addition, the amount of RS varies greatly depending on how food is prepared, cooked, and whether it is reheated (33). Cooked legumes, peas, and cooked and cooled starchy foods are high in RS. It may be wise to prepare food this way often, or to eat food prepared this way often. This finding, if confirmed in more studies, will have important clinical and public health implications.

## 5. Conclusion

Based on a nationally representative sample of U.S. adults, our study provides evidence that higher RS intake is associated with lower cancer and all-cause mortality, but not with CVD mortality. Future studies focusing on other populations with different food sources of RS and RS subtypes are needed to access the dose-response relationship and to improve global dietary recommendations for different populations.

## Data availability statement

The datasets presented in this study can be found in online repositories. The names of the repository/repositories and accession number(s) can be found below: https://wwwn.cdc.gov/nchs/nhanes/Default.aspx.

## Ethics statement

The studies involving human participants were reviewed and approved by NCHS Research Ethics Review Board. The patients/participants provided their written informed consent to participate in this study.

## Author contributions

XianL, JC, SY, and JW contributed to conception and design of the study. JW, XiaoL, and QL prepared the data for analyzes. MG and QL validated the data for analyzes. JW and XiaoL performed the formal analyzes and wrote the original draft. LL, CW, and RY reviewed and edited the draft. XianL, SY, and JC modified and provided reviews for the draft. All authors contributed to the article and approved the submitted version.
